# The syndromes of reduced sensitivity to thyroid hormone – the current state of art

**DOI:** 10.1186/1756-6614-8-S1-A5

**Published:** 2015-06-22

**Authors:** Anna Cyniak-Magierska

**Affiliations:** 1 Department of Endocrinology and Metabolic Diseases, Polish Mother’s Memorial Research Institute, The Medical University of Lodz, Lodz, Poland

## 

The clinical, laboratory, genetic and molecular characteristics of syndromes of reduced sensitivity to thyroid hormone are the subject of this abstract.

The syndrome of reduced sensitivity to thyroid hormone in the majority of cases is caused by point mutations in the thyroid hormone receptor β (T*Rβ*) gene. Before *TRβ* gene mutations were recognized, resistance to thyroid hormone (RTH) was subdivided on clinical basis into generalized, isolated pituitary and peripheral tissue. Nowadays this classification has a clinical usefulness, but it seems to have no logical etiologic grounds. The mutations in *TRβ* gene have been found in over 3000 individuals belonging to approximately 1000 families. While the clinical presentation is variable, the main features are: high serum FT_4_ and usually also FT_3_ concentrations, non-suppressed – sometimes slightly elevated serum thyrotropin (TSH), commonly a goiter. The majority of subjects have a near normal metabolic state, sometimes coexistence of clinical symptoms of thyroid hormones deficiency and excess takes place in one patient. Thus, delayed growth and bone maturation, and learning disabilities can be present along with hyperactive behavior and sinus tachycardia. Mental retardation was found in 3% of cases. Attention deficit hyperactivity disorder (ADHD) is also present in about half of patients with RTH syndrome.

In 15% of families with RTH symptoms no mutations in the *TRβ* gene were found. The term nonTR-RTH refers to this subgroup of individuals, clinically and biochemically identical with RTH caused by *TRβ* mutations.

Recently, mutations in *TRα1* gene have been described in two families. First nonsense mutation produces a truncated *TRα1* (E403X) that lacks the C-terminal α-helix. It has been identified in a 6 year-old girl with chronic constipation, and growth and developmental delay. Another family with *TRα1* gene mutation was described in 2012. In both cases, thyroid function tests were distinct from those in classical RTH with *TRβ* gene mutations. These patients had low serum T_4_, high T_3_, and very low rT_3_.

Two relatively novel syndromes presenting reduced sensitivity to thyroid hormone: membrane transport defect and thyroid hormone metabolism defect were described. This led to the broadening of the definition of reduced sensitivity to thyroid hormone to encompass all the defects that can interfere with the biological activity of a chemically intact hormone, secreted in normal amounts. Thyroid hormone cell membrane transporter defect (THCMTD) is caused by mutations in the *MCT8* gene. It is an X-linked defect. Mutations have 100% penetrance in males who manifest both neuropsychomotor impairment and characteristic thyroid test abnormalities (high serum T_3_, low rT_3_, low normal or reduced T_4_ with slightly elevated TSH level). The defect of the intracellular metabolism of thyroid hormones (THMD) is caused by mutations in the *SECISBP2* gene who is required for the synthesis of selenoproteins, including thyroid hormone deiodinases. It was described in 10 patients from 8 families.
